# Perspectives on Fracture Liaison Service in Austria: clinical and economic considerations

**DOI:** 10.3389/fendo.2024.1349579

**Published:** 2024-04-19

**Authors:** Roland Kocijan, Judith Haschka, Daniel Arian Kraus, Aaron Pfender, Stefan Frank, Jochen Zwerina, Martina Behanova

**Affiliations:** ^1^ Ludwig Boltzmann Institute of Osteology at Hanusch Hospital of Oesterreichische Gesundheitskasse (OEGK) and Allgemeine Unfallversicherungsanstalt (AUVA) Trauma Center Meidling, 1st Medical Department Hanusch Hospital, Vienna, Austria; ^2^ Metabolic Bone Diseases Unit, School of Medicine, Sigmund Freud University, Vienna, Austria; ^3^ AUVA Traumazentrum Wien, Standort Meidling Abteilung für Traumatologie, Vienna, Austria

**Keywords:** FLS, fracture, health care system, osteoporosis, prevention medicine

## Abstract

Osteoporosis is a widespread disease and affects over 500,000 people in Austria. Fragility fractures are associated with it and represent not only an individual problem for the patients, but also an enormous burden for the healthcare system. While trauma surgery care is well provided in Vienna, there is an enormous treatment gap in secondary prevention after osteoporotic fracture. Systematic approaches such as the Fracture Liaison Service (FLS) aim to identify patients with osteoporosis after fracture, to clarify diagnostically, to initiate specific therapy, and to check therapy adherence. The aim of this article is to describe the practical implementation and operational flow of an already established FLS in Vienna. This includes the identification of potential FLS inpatients, the diagnostic workup, and recommendations for an IT solution for baseline assessment and follow-up of FLS patients. We summarize the concept, benefits, and limitations of FLS and provide prospective as well as clinical and economic considerations for a city-wide FLS, managed from a central location. Future concepts of FLS should include artificial intelligence for vertebral fracture detection and simple IT tools for the implementation of FLS in the outpatient sector.

## 1 Introduction

In contrast to high traumatic fractures, which occur in the context of traffic, sports, recreational, and work accidents, fragility fractures happen after minimal trauma (e.g., falling from walking or standing) or even without any trauma in patients aged 50+ years. Thus, the cause of fracture is not the application of force, but reduced bone quality or quantity and, consequently, osteoporosis. The proportion of osteoporotic fractures in all fractures treated in the inpatient setting is 74.2% on average, meaning that three out of four fractures in the inpatient setting are due to osteoporosis rather than adequate trauma (1).

Fragility fractures represent a massive individual and socioeconomic problem. Traumatic care of fragility fractures is well ensured in Austria. However, current data from Austria show that the initiation of specific anti-osteoporotic therapy is weak. The proportion of women at high risk of fracture who did not receive treatment was 52% in 2019 (2, 3). The data on secondary prevention, i.e., treatment after an osteoporotic fracture, is even more disappointing. Only 1 in 10 men and less than 2 in 10 women receive appropriate therapy after a fragility fracture has occurred (4). Even after hip fracture, just 14% of patients receive anti-osteoporotic treatment (5). Thus, there is a huge “treatment gap” in the care of this high-risk population.

The typical locations of fragility fractures are hip, radius, vertebral bodies, humerus, and pelvis. These fractures are also referred to as “major osteoporotic fractures” (MOFs). Approximately 20%–30% of all people die within 1 year after hip fracture (6). This is comparable or even exceeds the 1-year mortality of malignancies or cardiovascular diseases (7, 8). The public health impact of hip fractures, as measured in disability-adjusted life years (DALYs), was 27 per 1,000 individuals. This equates to an average loss of 2.7% in healthy life expectancy (9). Additionally, the significant reduction in quality of life due to these fractures can be quantified using quality-adjusted life years (QALYs), a measure that reflects both the quality and the quantity of life gained or lost. Fragility fractures not only diminish DALYs but also negatively impact QALYs, underscoring the profound effects these injuries have on both the length and quality of life. Moreover, fragility fractures serve as prognostic factors for subsequent fractures as previous fracture history is associated with a significantly increased risk of any clinical fracture (10).

The risk of subsequent fractures doubles after a fracture (11), and remains high, especially within the first 2 years after fracture (12). For this reason, the terms “very high fracture risk” and “imminent fracture risk” have been introduced in recent years. A very high fracture risk comprises risk factors like recent fractures within the last year, multiple fractures and fractures during adequate treatment (13). Imminent fracture risk is defined as a very high risk for imminent fracture due to risk factors causing a significant short-term increase in fracture risk (14). Again, among others, a recent fracture is a particularly important factor indicating an imminent fracture risk (15).

Adequate treatment with anti-osteoporotic therapy after fragility fracture is therefore essential to reduce the risk of subsequent fracture, to decrease mortality, and to further reduce the burden on the healthcare system. The implementation of systematic approaches, such as the Fracture Liaison Service (FLS), play an important role in preventing subsequent fractures, resulting in huge savings in healthcare costs and improving the quality of life for patients.

## 2 Fragility fractures: facts and costs

The prevalence of osteoporosis in Austria is 552,000 or 5.5% of the total population (2). Data from Austria reported approximately 93,000 osteoporotic fractures in 2018. Estimations of the IOF (International Osteoporosis Foundation) suggested 110,000 new fragility fractures in 2019, equal to 300 fragility fractures per day (2). Thus, Austria is a “high-risk” country for osteoporotic fractures and, among the countries with the highest hip fracture incidence worldwide, surpassed only by Sweden and Denmark (16). In Vienna alone, 11,299 hip fractures were recorded in 2012–2016 (total in Austria, 57,623). This corresponds to an average of 2,260 hip fractures annually, or 6–7 hip fractures daily in Vienna (ÖVOS 2 project, data from the Austrian Sickness Funds, unpublished). Based on hip fracture data, it can be assumed that approximately 17%–20% of all osteoporotic fractures in Austria are treated in Vienna’s hospitals.

Data from the IOF show that fragility fractures in the setting of osteoporosis are among the most expensive conditions for our healthcare system. The estimated direct cost to the Austrian healthcare system is over EUR 157 million per year. This does not include rehabilitation, nursing care, or loss of work. The economic burden of fractures was EUR 1.3 billion in 2019 (3.4% of total national health expenditure) and has increased by EUR 501 million compared with 2010 (EUR 799 million in 2010) (2, 17). The most expensive fractures for our healthcare system are caused by hip fractures with nearly 77 million total annual costs (18).

## 3 Fracture Liaison Service

FLS represents a systematic, structured approach to prevent subsequent fractures. Evidence indicates that FLS also increases QALYs (19–21). FLS are implemented in hospitals with trauma surgery and are already established internationally in more than 50 countries. Based on the recommendations by Akkeson et al., FLS consists of (i) identification of fracture patients, (ii) evidence-based assessment including risk stratification and identification of secondary causes of osteoporosis, (iii) initiation of treatment, and (iv) improvement of long-term adherence to treatment (22). The interface between trauma surgery, osteoporosis specialists, and general practitioners is the FLS coordinator, who is mandatory for every FLS Center. This coordinator should come from the fields of medicine (general practitioners or specialists), nursing, or physical medicine.

**Figure 1:**
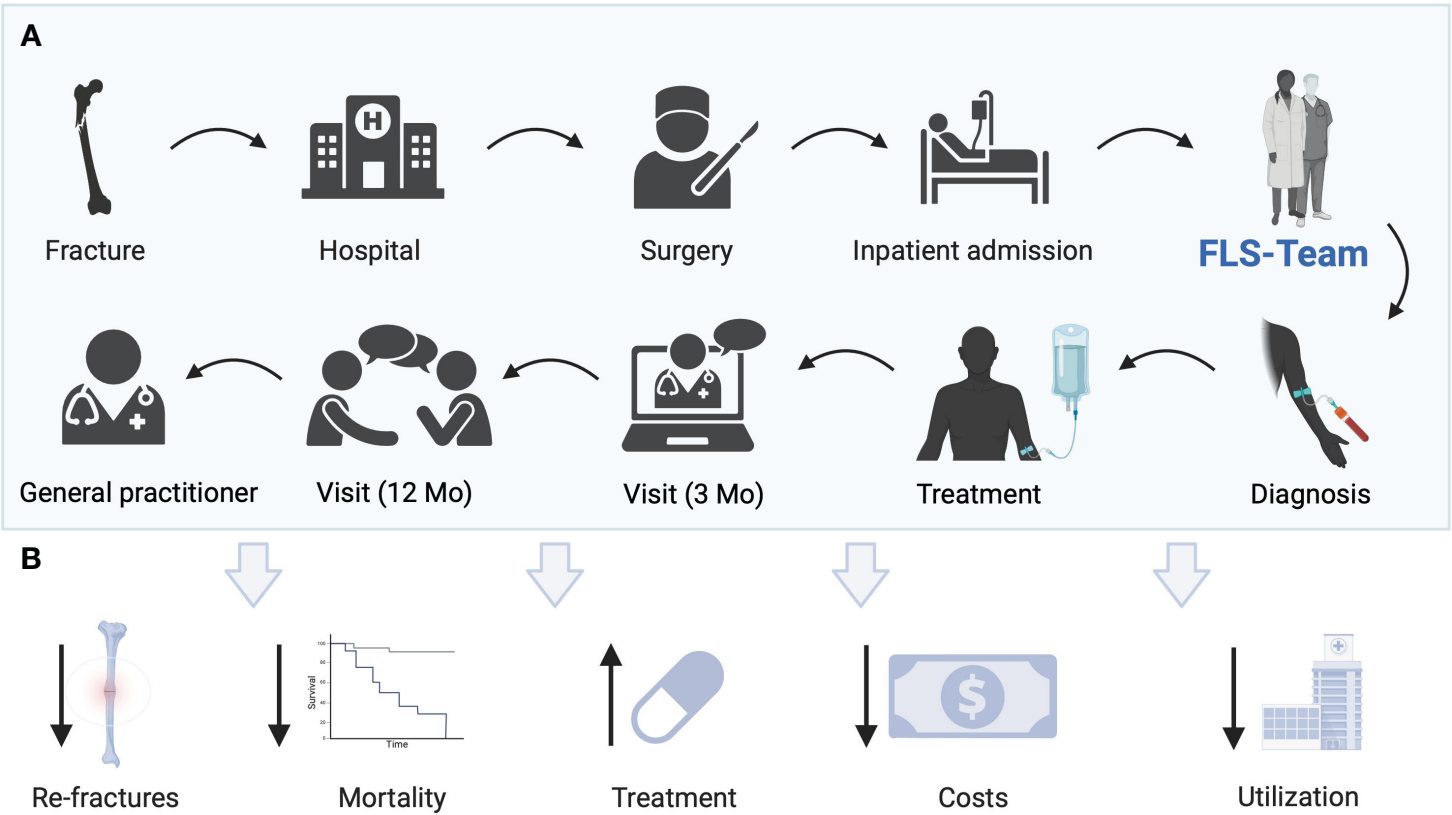
**(A)** Current concept of FLS at Hanusch Hospital Vienna. Patients with fractures are admitted to the hospital, operated on, or treated conservatively and admitted as inpatients. Patients are identified as possible patients with osteoporosis by the nursing team of the orthopedic surgery department. A request is made to the FLS team. A standardized blood draw is performed. The FLS team visits the patient. A treatment decision is made jointly. An optional visit takes place after 3 months. After 12 months, the final visit is carried out and the therapy adherence is checked. The patient is transferred for further care to the general practitioner’s office. **(B)** Potential benefits of FLS including reduction of re-fractures, mortality, costs, utilization of hospitals, and treatment gap. Created with BioRender.com.

According to the recommendations of the IOF initially as a first step, hip fractures should be managed by the FLS. Patients with hip fracture are of special importance in terms of morbidity and mortality as well as for the national economy. Hip fractures are treated exclusively on an inpatient basis and are therefore within easy reach of an FLS. Only after these fractures are well managed by the FLS should other fractures be treated in the inpatient setting. The third step involves outpatient fractures. Lastly, vertebral fractures should be included in the FLS (23). International FLS frameworks could raise treatment rates (24), reduce admissions to healthcare facilities, and improve long-term survival (25). The concept of FLS and the potential benefits are shown in **Figure 1**.

## 4 Implementation and operation of FLS in Vienna, Austria

In contrast to the above-mentioned recommendations by the IOF, not only hip fractures, but all MOFs have been included in our FLS from the beginning. This can be explained by the relatively low number of hip fractures in our hospital, compared to large trauma centers. Consequently, all inpatients (>50 years) who are admitted after hip, pelvis, radius, humerus, or clinical vertebral fracture are potential candidates for FLS. Other fractures (e.g., clavicula, tibia, skull, fingers, and toes) are not considered. Moreover, patients <50 years are not included in the FLS. However, in case of an unclear constellation of findings, an osteological consultation is performed. For big trauma centers, hip fractures should be the first fracture included in the FLS. From our point of view, in smaller hospitals with low number of fractures, all MOFs can be considered from the start, if resources allow it. Fractures treated on an outpatient basis as well as radiographic vertebral fractures are much more challenging and should only be included if all inpatient MOFs are already well integrated into the FLS.

### 4.1 Identification of FLS patients

Patients are identified as potential FLS patients after admission at the trauma surgery department by the nursing staff of the trauma surgery department during the transfer of duty. This is followed by a standardized blood draw the following day of admission based on the local recommendations (14). Moreover, parameters of bone metabolism including vitamin 25(OH)D, PTH, serum β-carboxy-terminal cross-linking telopeptide of type I collagen (CTX), and osteocalcin are determined. At the same time, the notification of the FLS team occurs. There are several options for this step. However, a digital solution seems to be the most suitable way (26) and was implemented therefore in our hospital. The FLS team is requested by a simple mouse click after the morning meeting. It is beneficial to adapt existing IT solutions [hospital information system (HIS)], as it has been done in our hospital.

### 4.2 FLS visit—diagnostic workup and treatment recommendations

Once the most important laboratory results are prevalent, a bedside visit with the patient is performed by the FLS coordinator. A detailed anamnesis and review of the current blood results are performed. Approval and treatment decisions are then made by the FLS physicians. If no further diagnostic workup is needed (applies to the majority of hip fractures), a treatment recommendation is made. Oral medications, osteo-anabolic medications, and denosumab are initiated on site. Intravenous bisphosphonates such as zoledronic acid may not be administered immediately after hip fracture, according to the current recommendations (14). The same applies to patients whose clinical or laboratory conditions prohibit administration in the inpatient setting (e.g., severe vitamin D deficiency and hypo- or hypercalcemia). In this case, patients are invited to an outpatient appointment 8–12 weeks later to receive the medication. Administration of iv medication can be performed by general practitioners, in the hospital’s own outpatient clinic or, as in our case, by an associated, outsourced outpatient clinic (health center). However, recent data suggest that an administration of zoledronic acid within 2 weeks is also useful (27). If further examinations are necessary, a second visit at the patient’s bedside or discussion of further procedures in the outpatient setting occurs 8–12 weeks after discharge. A final visit in the outpatient setting is scheduled 1 year after fracture.

The time required per patient for initial contact is approximately 20 min; for follow-up and final visits, it is 15 min. Intravenous administration of an osteoporosis drug takes approximately 45 min in an infusion outpatient clinic. Through the COVID-19 pandemic, it has been shown that follow-up visits can also be done very well by telemedicine (28). According to this calculation, approximately 10–15 fracture patients could be contacted, diagnosed, and cared for daily (6 h) by one FLS coordinator in presence or virtually via telecommunication. Since patients with hip fracture stay on average approximately 2 weeks in the inpatient setting (29), follow-up consultation is usually easy.

### 4.3 IT solution

A simple IT solution is mandatory for a successful FLS. Currently, no commercial software is available. Thus, the adaption of an existing software, connected to the hospital information systems (HIS), seems to be the most promising approach. **Supplementary Figure 1** shows the example of a “first-contact-form”. Relevant information on fracture risk (e.g., FRAX-recommended questions, co-medication, fracture history, etc.) is collected during the bedside visit. Similar contact forms are also available for follow-up visits.

The FLS interface includes a list of possible FLS patients, identified by the orthopedic surgery department and assigned to the FLS. They are then marked as patients with “osteoporotic fracture” and remain in the FLS, and thus in the IT system, for 52 weeks. Recent laboratory results are provided in the FLS interface. In addition, previously performed DXA measurements, imaging techniques of the spine (including x-rays of the chest and spine), and selected blood values within the past 2 years are also automatically included in the FLS interface (see **Supplementary Figure 2**).

The IT system is based on a traffic light system. Patients requested by the traumatology department are marked in red. Patients who have already been seen by the FLS nurse (including medical history and laboratory results) are marked yellow. Approval and treatment decisions are then made by the FLS physicians. After the visit is completed and the treatment decision has been made, the status is changed to green. The initial FLS assessment is thus completed. Follow-up visits are also incorporated in the HIS. These include questions on new risk factors, medication, and fractures as well as treatment adherence. Based on the IOF guideline, follow-ups are optional after 3 months and mandatory after 12 months after fracture.

These implementations ensure a complete history and transparency for further visits. Furthermore, all information can be transferred to patients´ discharge letters and a database for statistical evaluations on the feasibility and effectiveness of the FLS.

## 5 Discussion

### 5.1 Economic considerations

In the context of the escalating healthcare costs in Europe, osteoporotic fractures have emerged as a significant economic concern. In 2019, European countries reported total direct costs attributed to osteoporotic fractures amounting up to EUR 56.9 billion, of which incident fractures alone accounted for EUR 36.3 billion. However, the allocated budget for pharmacological interventions, which encompasses both diagnosis and treatment interventions, was relatively modest, standing at EUR 1.6 billion (30).

In 2019, Austria faced direct costs of incident fractures of EUR 833.5 million. Additionally, the indirect costs due to productivity losses and increased caregiver burden can be substantial (31), although more challenging to quantify. A systematic review has shown that these indirect costs could account for 2% to 50% of the total cost burden, depending on sample and methodology used (32). Summing up direct costs with long-term disability costs (EUR 468.1 million) and additional costs for pharmacological intervention (EUR 41.7 million) results in a total amount of EUR 1.3 billion for 1 year (2). Implementing more FLS across the country could reduce these direct costs by identifying and promptly treating individuals at elevated risk of subsequent fractures. Recent publications show that successful FLS programs lead to not only a risk reduction of re-fractures [hazard ratio (HR) 0.18–0.67 over 2–4 years] and mortality (HR 0.65 over 2 years) and increased initiation of therapy [relative risk (RR) 1.5–4.25], but also higher adherence to therapy (65%–88% at 1 year) (33). A decrease in recurrent fracture risk of approximately 10% can be achieved using FLS (34). Furthermore, FLS can notably decrease hospital admissions, surgical interventions, rehabilitation services, and extended care resulting from fracture.

The need for improvement of fracture care in Austria in terms of prevention of re-fractures is evident and the overall costs of osteoporotic fractures are increasing per capita. In 2019, the average direct costs of these fractures were EUR 151.8 per individual, which placed Austria at the sixth position among the EU27 + 2 countries in terms of highest costs per capita cost related to osteoporotic fractures (2). Osteoporotic fractures represented approximately 3.4% of Austria’s total healthcare expenditure in 2019 (2).

Globally, as the adoption of FLS grows, numerous countries have reported its effectiveness and efficacy (19, 35, 36). FLS is generally cost-effective for healthcare systems. A recent Dutch study further provided evidence that the implementation of FLS could lead to lifetime health-economic benefits. Patients with recent fractures managed by FLS had additional lifetime costs of 45 Euro compared to not being managed by FLS. However, FLS leads to an additional 0.11 QALYs, indicating an increase of quality of life for a certain time period (37).

Based on recently published data from Austria, the direct costs of one hip fracture are EUR 5,160. With 2,260 hip fractures in Vienna in 2018, this corresponds to costs of EUR 11,661,600 (18). A reduction by 10% would therefore correspond to savings of EUR 1,166,160 annually in direct costs for hip fractures only. Taking the total costs of osteoporotic fractures into account, the savings after implementation of an FLS program would be significantly higher. Although not directly comparable (different systems and different preconditions), data from a single center study in Finland showed that an FLS is inexpensive and accounted for only 1.3% of the annual total costs of all osteoporotic fractures in the study area (38).

Based on the System of Health Accounts (SHA), healthcare spendings in Austria are increasing and exceeded EUR 50 billion in 2022. This corresponds to 12.8% of the gross domestic product, being therefore among the highest worldwide.

While the total public healthcare expenditure was EUR 38.5 billion in 2021, only 4.8 billion of this amount was spent on preventive measures. The majority was spent on inpatient and outpatient healthcare.

However, despite the clear advantages that FLS offers in terms of secondary fracture prevention and the potential for economic savings, their availability in Austria remains limited. The only IOF-certified FLS is located in Hanusch hospital in Vienna, although there are some attempts to provide FLS-alike procedures in some hospitals across the country. By expanding the reach and certification of FLS across more hospitals, there is a promising opportunity to reduce the economic burden associated with osteoporotic fractures. The evidence shows that an investment in FLS can lead to both improved healthcare outcomes and significant economic savings (20).

### 5.2 Outlook

In addition to the known benefits of FLS, there are also some limitations and difficulties in setting up an FLS. Although the IOF makes recommendations, healthcare systems vary from country to country. The basic prerequisite for an FLS is the commitment of the healthcare provider and those involved. Setting up an FLS initially costs money before it saves money. The positive results, including the reduction in fracture risk, are not expected in the short term, but only materialize over time. Human resources are the key factor for an FLS. At least one FLS coordinator is required for a successful FLS. In the best case, a multidisciplinary team takes care of the FLS. After the implementation of hip fractures, all MOFs should be included in the FLS. Once this is done, outpatients and radiographic vertebral fractures should also be managed. At present, only a few FLSs have already implemented this due to the logistical effort involved. A defined workflow, collaboration between orthopedic surgery and internal medicine, and simple IT solutions for data collection and follow-up of patients, as well as the identification of outpatients, could improve FLS systems worldwide. In addition, the use of artificial intelligence (AI) for FLS is currently being researched and appears to be promising.

#### 5.2.1 Outpatients

Out of 93,000 osteoporotic fractures in Austria annually, 55,707 were treated in an outpatient care setting (1). However, including outpatients into the FLS is more complicated by facing difficulties in terms of organization, working hours, and interdisciplinary setting. Consequently, since most FLS programs focus on inpatients, the majority of, e.g., radial fractures are missed. Awareness campaigns for outpatient clinics are therefore needed. An electronic approach would be providing QR-based information on disease, fracture risk assessment, and contact information for further assessment and treatment options.

#### 5.2.2 AI

One of the most challenging osteoporotic fractures are vertebral fractures, as 65% to 75% were asymptomatic and only one-third of vertebral deformities come to medical attention (39, 40). Besides that, diagnosing vertebral fractures remains challenging as well. A prospective analysis from Geneva et al. showed, that only about one-third of vertebral fractures were reported on lateral spine and chest radiographs by the radiologist (41). The application of AI for opportunistic vertebral fracture detection on radiographs or CT scans could be helpful in identifying vertebral fractures and bringing them to clinical attention by importing AI results into the FLS workflow (42, 43).

#### 5.2.3 Home nursing

Parenteral therapies (intravenous or subcutaneous) that were prescribed during the FLS visit but not implemented in the inpatient setting due to contraindications (e.g., severe vitamin D insufficiency and hypocalcemia) could be administered at the patient’s home within the framework of “home nursing”. Nurses visit the patients on site and infuse the medication. This service should be reserved for immobilized or bedridden patients for whom a trip to the outpatient clinic is not possible.

#### 5.2.4 Tools

Pocket cards based on the FLS standard operating procedure (SOP) including QR codes and appointment cards with the date of the follow-up visit, the scheduled medication, and the outpatient clinic contact information might be useful tools for staff and patients, respectively.

In the UK, national toolkits and educational programs are available to improve secondary prevention after osteoporotic fracture (44). In Spain, a national hip fracture registry was established to improve assessment and to close the treatment gap (45). A guide to fall prevention at home has already been implemented in Spain and Canada (44).

#### 5.2.5 City-wide FLS

**Figure 2:**
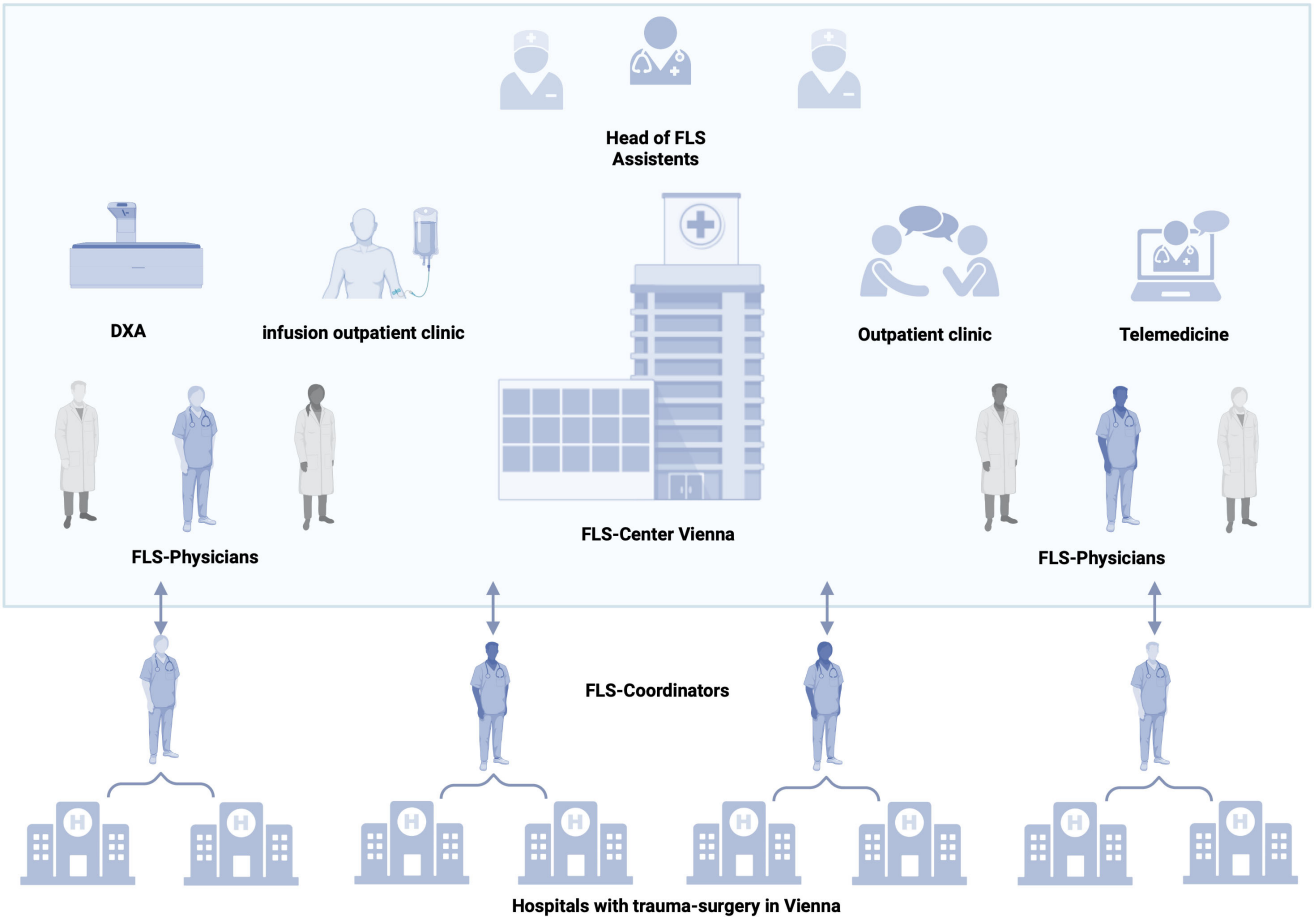
Concept of a city-wide FLS. Head, assistant staff, FLS physicians, and nurses are on site. The center includes outpatient rooms for follow-up visits, telemedicine, an infusion outpatient clinic for therapy administration, and special diagnostics. Each FLS coordinator supervises two hospitals with departments for trauma surgery. After the ward round and documentation, consultation with the physicians in the center takes place and a therapy decision is made. Created with BioRender.com.

In Vienna, eight trauma departments take care of the initial treatment of fracture patients. A centralized FLS could serve all eight hospitals in the inpatient setting and then follow up patients in the outpatient setting. A city-wide FLS in Vienna would prevent secondary fractures, reduce morbidity and mortality of patients with osteoporosis, and relieve hospitals and the healthcare system. In addition, it would make a significant contribution to the concept of prevention. A central lead position is essential to provide standardized care. The FLS team could visit and oversee all trauma surgery departments. On the one hand, this would optimize patient care; on the other hand, it would provide the opportunity to regularly evaluate and improve the central FLS. A perspective for a city-wide FLS is shown in **Figure 2**.

A city-wide FLS would require the following:

(i) A head of FLS, responsible for the coordination of the FLS, continuous improvement of the FLS, and communication with stakeholders.
(ii) FLS coordinator (one per two hospitals), who will perform inpatient visits, FLS follow-up visits via telemedicine, data collection, and communication with hospital staff.
(iii) Physicians with focus on osteology (one per two hospitals) for decision-making on further diagnostic steps and therapy, outpatient assessments (follow-up visits after 3 months and after 1 year, respectively), and linking patients to the general practice sector after completion of the year.
(iv) For the outpatient setting, a certified healthcare nurse is needed for medication administration and patient education.
(v) Assistant staff for registration and appointment coordination.

## 6 Conclusion

In conclusion, fragility fractures are common in Austria and represent not only an individual problem, but also an economic burden. Secondary fracture prevention, in the context of FLS, reduces fractures and mortality and relieves the healthcare system. A centrally controlled FLS for the eight hospitals in Vienna with trauma fracture care could thus make a significant contribution to healthcare. Internationally established approaches as well as digital solutions should be implemented in Austria in the future.

## Data availability statement

The original contributions presented in the study are included in the article/**Supplementary Material**. Further inquiries can be directed to the corresponding author.

## Ethics statement

The studies involving humans were approved by Ethikkommission der Stadt Wien Thomas-Klestil-Platz 8/21030, WienEK-19-220-VK. The studies were conducted in accordance with the local legislation and institutional requirements. Written informed consent for participation was not required from the participants or the participants’ legal guardians/next of kin in accordance with the national legislation and institutional requirements.

## Author contributions

RK: Writing – original draft, Conceptualization. JH: Writing – original draft. DK: Writing – original draft. AP: Writing – review & editing, Visualization. SF: Writing – review & editing. JZ: Writing – review & editing, Supervision. MB: Writing – original draft, Data curation.

## Supplementary material

Supplementary Figure 1 First-contact form. Clinical data on fracture risk (including those for FRAX) are assessed by the FLS nurse during the bedside visit using a laptop or tablet.

Supplementary Figure 2 FLS interface implemented in the hospital information system (HIS). Orange arrow indicates possible FLS patients, identified by the orthopedic surgery department and assigned to the FLS. They are marked as “osteoporotic fracture” patients (gray arrow) and remain in the FLS for 52 weeks. Blue arrow indicates the status; red dot: patient assigned, not seen so far (yellow would indicate seen by an FLS nurse, not approved by an FLS physician; green would indicate seen by an FLS nurse and approved by the physician, including diagnostic and therapeutic decision); green arrows: previously performed DXA scans, x-rays, and other imaging procedures detecting vertebral fractures are automatically shown in the interface.

## References

[1] Hummer M Hlava A Birner A. Epidemiologie osteoporotischer Fragilitätsfrakturen (2020). Available at: https://jasmin.goeg.at/1553/ (Accessed November 24, 2023).

[2] Willers C Norton N Harvey NC Jacobson T Johansson H Lorentzon M. Osteoporosis in Europe: a compendium of country-specific reports. Arch Osteoporos. (2022) 17:23. doi: 10.1007/s11657-021-00969-8 35079919 PMC8789736

[3] Kanis JA Borgström F Compston J Dreinhöfer K Nolte E Jonsson L. SCOPE: a scorecard for osteoporosis in Europe. Arch Osteoporos. (2013) 8:144. doi: 10.1007/s11657-013-0144-1 24030479 PMC3880480

[4] Malle O Borgstroem F Fahrleitner-Pammer A Svedbom A Dimai SV Dimai HP. Mind the gap: Incidence of osteoporosis treatment after an osteoporotic fracture – results of the Austrian branch of the International Costs and Utilities Related to Osteoporotic Fractures Study (ICUROS). Bone. (2021) 142:115071. doi: 10.1016/j.bone.2019.115071 31593822

[5] Behanova M Reichardt B Stamm T Zwerina J Klaushofer K Kocijan R. Treatment effects of bisphosphonates and denosumab on survival and refracture from real-world data of hip-fractured patients. Calcif Tissue Int. (2019) 105(6):630–41. doi: 10.1007/s00223-019-00611-3 31531720

[6] Brozek W Reichardt B Kimberger O Zwerina J Dimai HP Kritsch D. Mortality after hip fracture in Austria 2008–2011. Calcif Tissue Int. (2014) 95:257–66. doi: 10.1007/s00223-014-9889-9 24989776

[7] Cowling TE Bellot A Boyle J Walker K Kuryba A Galbraith S. One-year mortality of colorectal cancer patients: development and validation of a prediction model using linked national electronic data. Br J Cancer. (2020) 123:1474–80. doi: 10.1038/s41416-020-01034-w PMC7652941 32830202

[8] Ye Q Zhang J Ma L. Predictors of all-cause 1-year mortality in myocardial infarction patients. Med (Baltimore). (2020) 99:e21288. doi: 10.1097/MD.0000000000021288 PMC7373524 32702922

[9] Papadimitriou N Tsilidis KK Orfanos P Benetou V Ntzani EE Soerjomataram I. Burden of hip fracture using disability-adjusted life-years: a pooled analysis of prospective cohorts in the CHANCES consortium. Lancet Public Health. (2017) 2:e239–46. doi: 10.1016/S2468-2667(17)30046-4 29253489

[10] Kanis JA Johansson H McCloskey EV Liu E Åkesson KE Anderson FA. Previous fracture and subsequent fracture risk: a meta-analysis to update FRAX. Osteoporos Int. (2023) 34(12):2027–45. doi: 10.1007/s00198-023-06870-z 37566158 PMC7615305

[11] Johansson H Siggeirsdóttir K Harvey NC Odén A Gudnason V McCloskey E. Imminent risk of fracture after fracture. Osteoporos Int J Establ Result Coop Eur Found Osteoporos Natl Osteoporos Found USA. (2017) 28:775–80. doi: 10.1007/s00198-016-3868-0 PMC5338733 28028554

[12] Toth E Banefelt J Åkesson K Spångeus A Ortsäter G Libanati C. History of previous fracture and imminent fracture risk in Swedish women aged 55 to 90 years presenting with a fragility fracture. J Bone Miner Res. (2020) 35:861–8. doi: 10.1002/jbmr.3953 PMC9328134 31914206

[13] Camacho PM Petak SM Binkley N Diab DL Eldeiry LS Farooki A. American association of clinical Endocrinologists/American college of endocrinology clinical practice guidelines for the diagnosis and treatment of postmenopausal osteoporosis-2020 update. Endocr Pract Off J Am Coll Endocrinol Am Assoc Clin Endocrinol. (2020) 26:1–46. doi: 10.4158/GL-2020-0524SUPPL 32427503

[14] DVO-Leitlinien. Available at: https://www.lmu-klinikum.de/osz/downloads-und-links/dvo-leitlinien/a98431f345669bc0 (Accessed November 16, 2023).

[15] Gregson CL Armstrong DJ Bowden J Cooper C Edwards J Gittoes NJL. UK clinical guideline for the prevention and treatment of osteoporosis. Arch Osteoporos. (2022) 17:58. doi: 10.1007/s11657-022-01061-5 35378630 PMC8979902

[16] Kanis JA Odén A McCloskey EV Johansson H Wahl DA Cooper C. A systematic review of hip fracture incidence and probability of fracture worldwide. Osteoporos Int J Establ Result Coop Eur Found Osteoporos Natl Osteoporos Found USA. (2012) 23:2239–56. doi: 10.1007/s00198-012-1964-3 PMC3421108 22419370

[17] Svedbom A Hernlund E Ivergård M Compston J Cooper C Stenmark J. Osteoporosis in the European Union: a compendium of country-specific reports. Arch Osteoporos. (2013) 8:137. doi: 10.1007/s11657-013-0137-0 24113838 PMC3880492

[18] Muschitz C Hummer M Grillari J Hlava A Birner AH Hemetsberger M. Epidemiology and economic burden of fragility fractures in Austria. Osteoporos Int J Establ Result Coop Eur Found Osteoporos Natl Osteoporos Found USA. (2022) 33:637–47. doi: 10.1007/s00198-021-06152-6 PMC8497183 34622302

[19] Wu C-H Kao I-J Hung W-C Lin S-C Liu H-C Hsieh M-H. Economic impact and cost-effectiveness of fracture liaison services: a systematic review of the literature. Osteoporos Int. (2018) 29:1227–42. doi: 10.1007/s00198-018-4411-2 29460102

[20] Pinedo-Villanueva R Burn E Maronga C Cooper C Javaid MK. Expected benefits and budget impact from a microsimulation model support the prioritization and implementation of fracture liaison services. J Bone Miner Res. (2023) 38:499–511. doi: 10.1002/jbmr.4775 36662166

[21] Naranjo A Prieto-Alhambra D Sánchez-Martín J Pérez-Mitru A Brosa M. Cost-effectiveness analysis of fracture liaison services compared with standard of care in the secondary prevention of fragility fractures in Spain. Clin Outcomes Res CEOR. (2022) 14:249–64. doi: 10.2147/CEOR.S350790 PMC9041144 35492806

[22] Åkesson K Marsh D Mitchell PJ McLellan AR Stenmark J Pierroz DD. Capture the Fracture: a Best Practice Framework and global campaign to break the fragility fracture cycle. Osteoporos Int. (2013) 24:2135–52. doi: 10.1007/s00198-013-2348-z PMC3706734 23589162

[23] Capture the Fracture®. International Osteoporosis Foundation. Available at: https://www.osteoporosis.foundation/capture-the-fracture (Accessed November 14, 2023).

[24] Hoggard TM Jeray KJ. Osteoporosis management in the United States. OTA Int. (2022) 5:e184. doi: 10.1097/oi9.0000000000000184 35949495 PMC9359008

[25] Ruggiero C Baroni M Talesa GR Cirimbilli A Prenni V Bubba V. The interdisciplinary fracture liaison service improves health-related outcomes and survival of older adults after hip fracture surgical repair. Arch Osteoporos. (2022) 17:135. doi: 10.1007/s11657-022-01171-0 36251126 PMC9576663

[26] Javaid MK Pinedo-Villanueva R Shah A Mohsin Z Hiligsmann M Motek-Soulié A. The Capture the Fracture® Partnership: an overview of a global initiative to increase the secondary fracture prevention care for patient benefit. Osteoporos Int J Establ Result Coop Eur Found Osteoporos Natl Osteoporos Found USA. (2023) 34:1827–35. doi: 10.1007/s00198-023-06759-x PMC10579122 37418152

[27] Education Resource Center - American Society for Bone and Mineral Research. Available at: https://www.asbmr.org/education-resources/education-resource-center (Accessed November 15, 2023).

[28] English S Coyle L Bradley S Wilton W Cordner J Dempster R. Virtual fracture liaison clinics in the COVID era: an initiative to maintain fracture prevention services during the pandemic associated with positive patient experience. Osteoporos Int. (2021) 32:1221–6. doi: 10.1007/s00198-021-05882-x PMC7882233 33585952

[29] Ireland AW Kelly PJ Cumming RG. Total hospital stay for hip fracture: measuring the variations due to pre-fracture residence, rehabilitation, complications and comorbidities. BMC Health Serv Res. (2015) 15:17. doi: 10.1186/s12913-015-0697-3 25609030 PMC4308914

[30] Kanis JA Norton N Harvey NC Jacobson T Johansson H Lorentzon M. SCOPE 2021: a new scorecard for osteoporosis in Europe. Arch Osteoporos. (2021) 16:82. doi: 10.1007/s11657-020-00871-9 34080059 PMC8172408

[31] Yeh EJ Rajkovic-Hooley O Silvey M Ambler WS Milligan G Pinedo-Villanueva R. Impact of fragility fractures on activities of daily living and productivity in community-dwelling women: a multi-national study. Osteoporos Int J Establ Result Coop Eur Found Osteoporos Natl Osteoporos Found USA. (2023) 34:1751–62. doi: 10.1007/s00198-023-06822-7 PMC10511617 37335332

[32] Ruiz-Adame M Correa M. A systematic review of the indirect and social costs studies in fragility fractures. Osteoporos Int J Establ Result Coop Eur Found Osteoporos Natl Osteoporos Found USA. (2020) 31:1205–16. doi: 10.1007/s00198-020-05319-x 32002572

[33] Walters S Khan T Ong T Sahota O. Fracture liaison services: improving outcomes for patients with osteoporosis. Clin Interv Aging. (2017) 12:117–27. doi: 10.2147/CIA.S85551 PMC5237590 28138228

[34] Inderjeeth CA Raymond WD Geelhoed E Briggs AM Oldham D Mountain D. Fracture liaison service utilising an emergency department information system to identify patients effectively reduce re-fracture rate is cost-effective and cost saving in Western Australia. Australas J Ageing. (2022) 41:e266–75. doi: 10.1111/ajag.13107 PMC9545318 35811331

[35] Li N Hiligsmann M Boonen A van Oostwaard MM de Bot RTAL Wyers CE. The impact of fracture liaison services on subsequent fractures and mortality: a systematic literature review and meta-analysis. Osteoporos Int. (2021) 32:1517–30. doi: 10.1007/s00198-021-05911-9 PMC8376729 33829285

[36] Wu C-H Tu S-T Chang Y-F Chan D-C Chien J-T Lin C-H. Fracture liaison services improve outcomes of patients with osteoporosis-related fractures: A systematic literature review and meta-analysis. Bone. (2018) 111:92–100. doi: 10.1016/j.bone.2018.03.018 29555309

[37] Li N van den Bergh JP Boonen A Wyers CE Bours SPG Hiligsmann M. Cost-effectiveness analysis of fracture liaison services: a Markov model using Dutch real-world data. Osteoporos Int. (2023) 35(2):293–307. doi: 10.1007/s00198-023-06924-2 37783759 PMC10837229

[38] Lüthje P Nurmi-Lüthje I Tavast N Villikka A Kataja M. Evaluation of minimal fracture liaison service resource: costs and survival in secondary fracture prevention—a prospective one-year study in South-Finland. Aging Clin Exp Res. (2021) 33:3015–27. doi: 10.1007/s40520-021-01826-x PMC8595226 33811622

[39] Fink HA Milavetz DL Palermo L Nevitt MC Cauley JA Genant HK. What proportion of incident radiographic vertebral deformities is clinically diagnosed and vice versa? J Bone Miner Res. (2005) 20:1216–22. doi: 10.1359/JBMR.050314 15940375

[40] Cooper C Atkinson EJ O’Fallon WM Melton LJ. Incidence of clinically diagnosed vertebral fractures: a population-based study in Rochester, Minnesota, 1985-1989. J Bone Miner Res Off J Am Soc Bone Miner Res. (1992) 7:221–7. doi: 10.1002/jbmr.5650070214 1570766

[41] Casez P Uebelhart B Gaspoz J-M Ferrari S Louis-Simonet M Rizzoli R. Targeted education improves the very low recognition of vertebral fractures and osteoporosis management by general internists. Osteoporos Int J Establ Result Coop Eur Found Osteoporos Natl Osteoporos Found USA. (2006) 17:965–70. doi: 10.1007/s00198-005-0064-z 16758137

[42] Nicolaes J Liu Y Zhao Y Huang P Wang L Yu A. External validation of a convolutional neural network algorithm for opportunistically detecting vertebral fractures in routine CT scans. Osteoporos Int. (2023) 35(1):143–52. doi: 10.1007/s00198-023-06903-7 37674097 PMC10786735

[43] Shen L Gao C Hu S Kang D Zhang Z Xia D. Using artificial intelligence to diagnose osteoporotic vertebral fractures on plain radiographs. J Bone Miner Res Off J Am Soc Bone Miner Res. (2023) 38:1278–87. doi: 10.1002/jbmr.4879 37449775

[44] Chesser TJS Javaid MK Mohsin Z Pari C Belluati A Contini A. Overview of fracture liaison services in the UK and Europe: standards, model of care, funding, and challenges. OTA Int. (2022) 5:e198. doi: 10.1097/OI9.0000000000000198 35949498 PMC9359010

[45] Montoya-Garcia MJ Carbonell-Abella C Cancio-Trujillo JM Moro-Álvarez MJ Mora-Fernández J Izquierdo-Avino R. Spanish National Registry of Major Osteoporotic Fractures (REFRA) seen at Fracture Liaison Services (FLS): objectives and quality standards. Arch Osteoporos. (2022) 17:138. doi: 10.1007/s11657-022-01174-x 36318373 PMC9626427

