# Supplementary figures and images for: Perspectives on Fracture Liaison Service in Austria: clinical and economic considerations
Source: Front Endocrinol (Lausanne). 2024 Apr 19;15:1349579. doi: 10.3389/fendo.2024.1349579 (PMC11066262; doi:10.3389/fendo.2024.1349579)

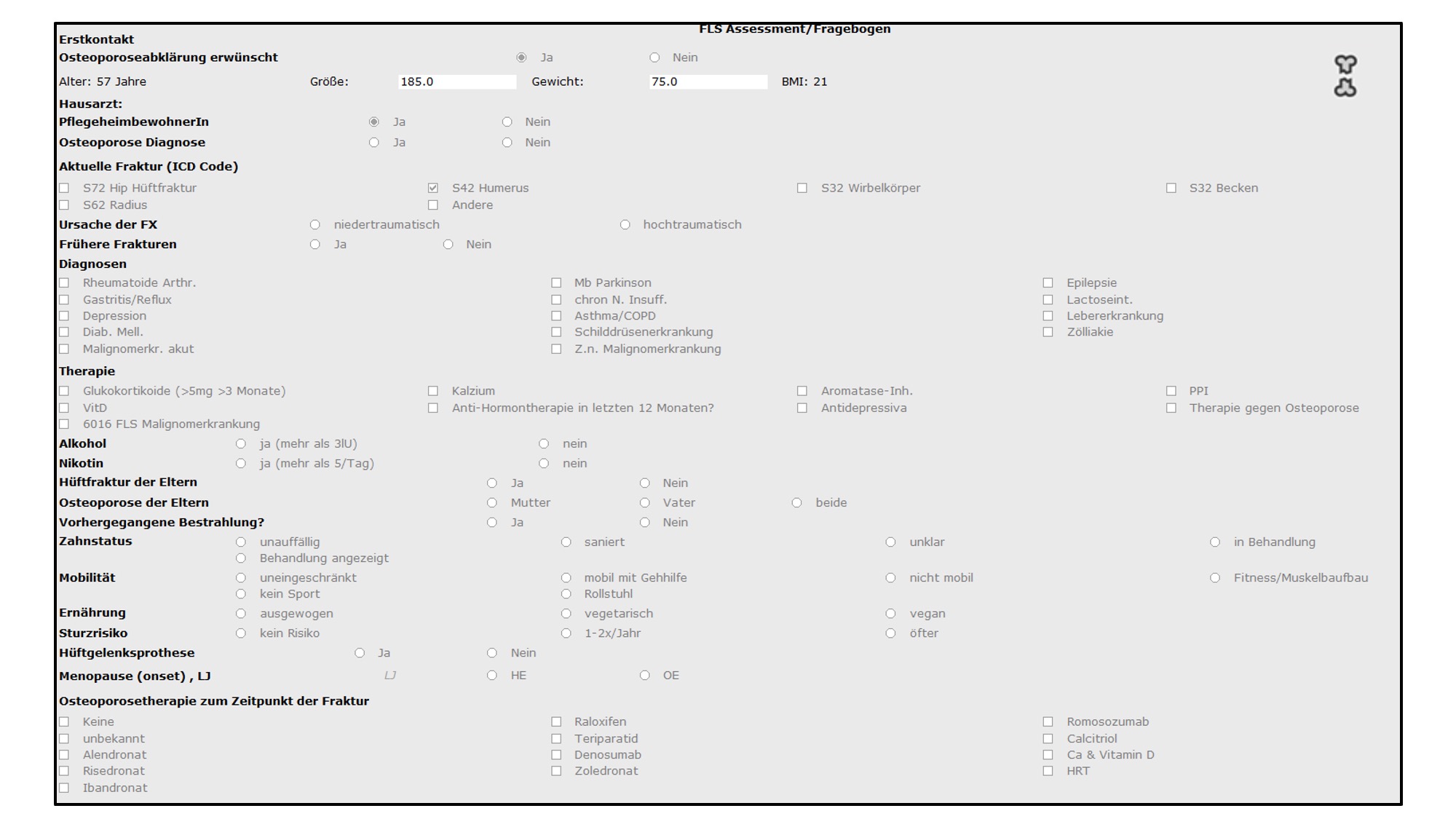

Supplement: Supplementary Figure 1 — First-contact form. Clinical data on fracture risk (including those for FRAX) are assessed by the FLS nurse during the bedside visit using a laptop or tablet. [file Image_1.jpeg]

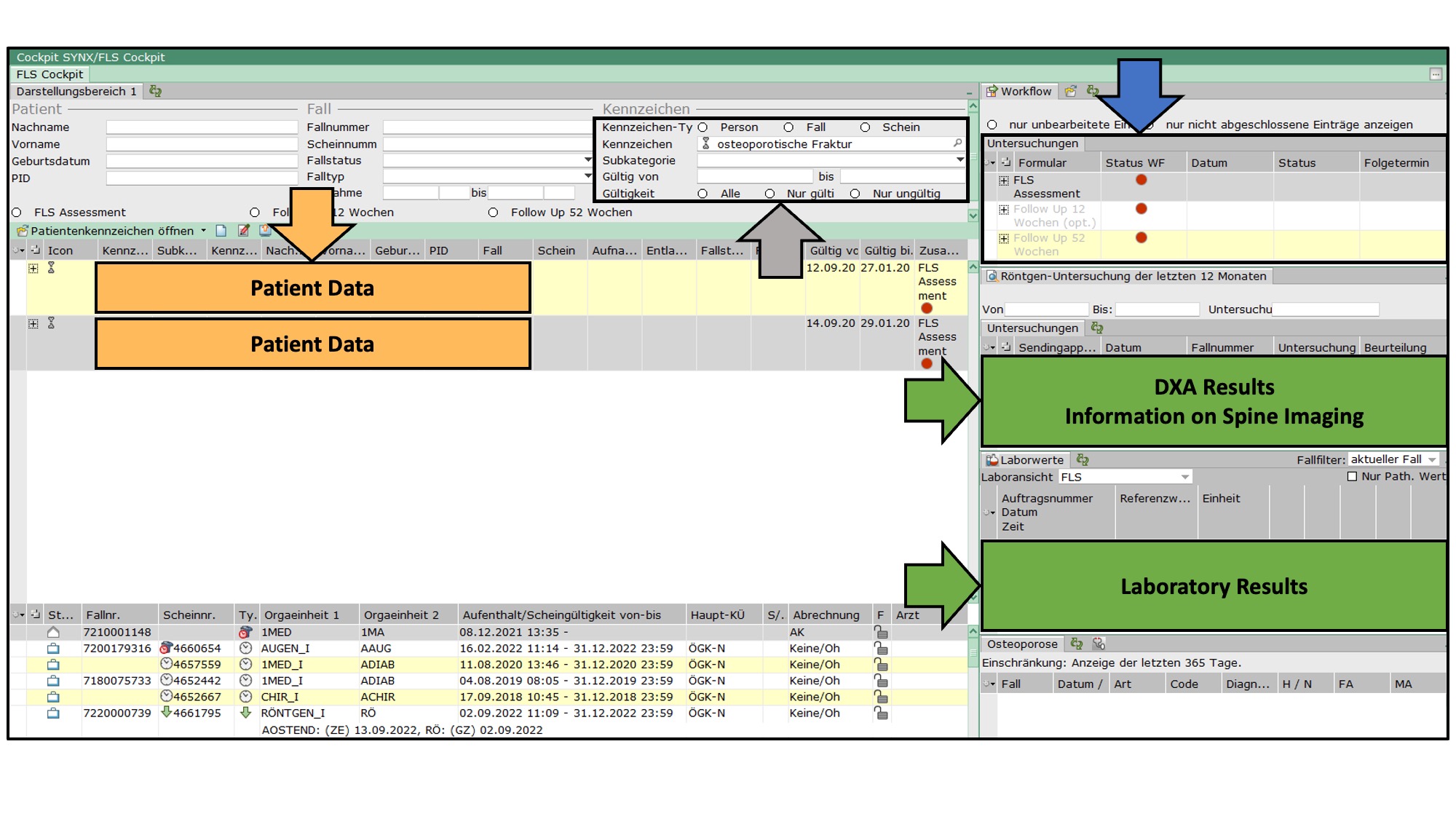

Supplement: Supplementary Figure 2 — FLS interface implemented in the hospital information system (HIS). Orange arrow indicates possible FLS patients, identified by the orthopedic surgery department and assigned to the FLS. They are marked as “osteoporotic fracture” patients (gray arrow) and remain in the FLS for 52 weeks. Blue arrow indicates the status; red dot: patient assigned, not seen so far (yellow would indicate seen by an FLS nurse, not approved by an FLS physician; green would indicate seen by an FLS nurse and approved by the physician, including diagnostic and therapeutic decision); green arrows: previously performed DXA scans, x-rays, and other imaging procedures detecting vertebral fractures are automatically shown in the interface. [file Image_2.jpeg]
